# The Janus Head of T Cell Aging – Autoimmunity and Immunodeficiency

**DOI:** 10.3389/fimmu.2013.00131

**Published:** 2013-06-04

**Authors:** Jörg J. Goronzy, Guangjin Li, Zhen Yang, Cornelia M. Weyand

**Affiliations:** ^1^Division of Immunology and Rheumatology, Department of Medicine, Stanford University School of Medicine, Stanford, CA, USA; ^2^Department of Medicine, Palo Alto Veteran Administration Health Care System, Palo Alto, CA, USA

**Keywords:** immunosenescence, autoimmunity, inflammation, pathogenesis, DNA damage response, T cell receptor signaling, rheumatoid arthritis, giant cell arteritis

## Abstract

Immune aging is best known for its immune defects that increase susceptibility to infections and reduce adaptive immune responses to vaccination. In parallel, the aged immune system is prone to autoimmune responses and many autoimmune diseases increase in incidence with age or are even preferentially encountered in the elderly. Why an immune system that suboptimally responds to exogenous antigen fails to maintain tolerance to self-antigens appears to be perplexing. In this review, we will discuss age-associated deviations in the immune repertoire and the regulation of signaling pathways that may shed light on this conundrum.

## Introduction

**Figure 1:**
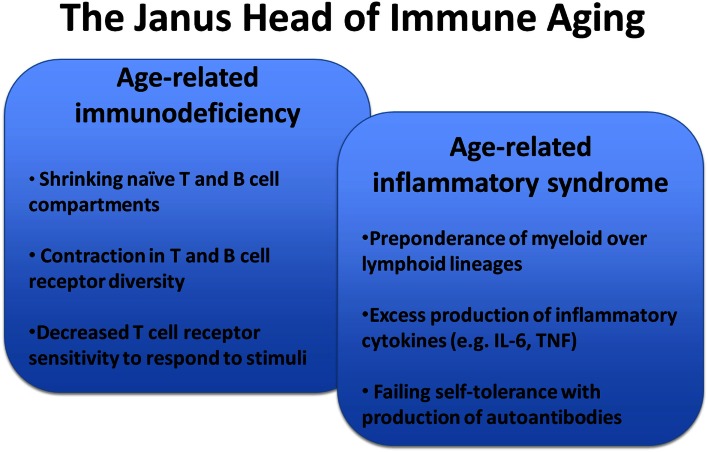
**Key features of immunosenescence**. Immune aging is a multifaceted process that generates a complex presentation of impaired adaptive immune responses, constitutive low-grade inflammation, and autoimmunity.

With increasing age, the ability of the immune system to protect against infection or to mount adaptive immune responses after vaccinations declines (Thompson et al., 2003; Jefferson et al., 2005; Nichol et al., 2007; Targonski et al., 2007). The mechanisms of this immune dysfunction are multidimensional. However, at the core is an inability of T cells to translate recognition of antigenic peptide in the context of the appropriate HLA molecule into productive T cell activation, clonal expansion, and differentiation into effector cells that provide help for B cells to differentiate or that exert effector function (Weng, 2006). We have recently shown that this dysfunctionality is, in part, conferred by the activation of negative feedback mechanisms in T cell signaling that raise the T cell receptor activation threshold or that negatively control sustained activation and lead to the early termination of differentiation pathways (Goronzy et al., 2012). While immune aging is perceived as a loss in effectiveness, it also brings along increased inflammation and a higher susceptibility to develop autoimmune diseases (Figure 1) (Goronzy and Weyand, 2012). Increased titers of autoantibodies, such as rheumatoid factor or antinuclear antibodies after the age of 60 years are well recognized and often are an indicator of increased autoreactivity without disease relevance (Moulias et al., 1984; Ruffatti et al., 1990a,b). More importantly, many autoimmune diseases show bimodal age of onset distributions with the first peak in young adulthood and the second peak in older age; or, alternatively, they occur in late adulthood and then increase in incidence with age (Cooper and Stroehla, 2003; Crowson et al., 2011). Typical examples are rheumatoid arthritis that occurs in females preferentially after menopause with steadily increasing incidence in the sixth and seventh decades of age (Doran et al., 2002). Even more dramatically, polymyalgia rheumatica and giant cell arteritis (GCA) are only found in individuals older than 50 years and the risk of developing GCA continues to increase into the 1980s (Weyand and Goronzy, 2003; Kermani et al., 2010; Mohan et al., 2011).

In the current paradigm, autoimmunity develops when self-reactive T and B cells recognize a self-antigen and differentiate into memory and effector cells. Recognition of self-antigens by self-reactive T cells is by virtue of the thymic selection process a low affinity interaction, which should be most affected by the negative regulatory signaling pathways, deviations of which we have discovered to occur in aging T cells (Goronzy et al., 2012). The co-occurrence of declining immunocompetence and increasing autoimmune susceptibility therefore appears to be contradictory. In this review, we will discuss possible models that overcome this apparent paradox and ultimately explore the hypothesis that the same defects that account for the decreased ability to generate protective immune responses also contribute to the increased risk of autoimmunity.

## Peripheral Repertoire Selection with Age as a Risk Factor for Autoimmunity

The T cell receptor repertoire is essentially fully established in the first 20 years of life when new T cells are generated in the thymus. Thymic activity is the highest immediately after birth and then steadily declines. It is debatable whether in healthy individuals any thymic T cell generation occurs after the age of 20 years. Ongoing thymic activity is necessary to maintain a naïve T cell compartment in the mouse where the decline in thymic output is responsible for the eventual loss in naïve T cells (den Braber et al., 2012). In contrast, a fundamentally different rule appears to apply to T cell homeostasis in men where virtually all naïve T cell generation in humans after the age of 20 arises from self-renewal of the existing T cell pool (den Braber et al., 2012). This model is consistent with our recent *in silico* simulation of T cell homeostasis with progressive age (Johnson et al., 2012). The simulation was most consistent with a model in which thymic output was neither necessary nor beneficial for maintaining a diverse naïve T cell repertoire with age. In fact, virtual rejuvenation of thymic activity was not able to prevent or restore a contraction in diversity. Most factual observations on human T cell homeostasis are consistent with or explained by these virtual data (Goronzy and Weyand, 2005). The size of the naïve CD4 T cell compartment in humans only moderately shrinks with aging and the T cell receptor diversity is very well maintained up to the eighth decade of life when it abruptly collapses (Czesnikiewicz-Guzik et al., 2008). In our model, this abrupt contraction is best reproduced by cumulative changes in growth behavior and peripheral selection. In bone marrow transplant studies, reactivation of the thymus and repopulation of the peripheral T cell compartment was no longer achievable in the majority of individuals older than 40–50 years (Hakim et al., 2005). Finally, there is no evidence for compensatory increase in peripheral T cell turnover between the age of 20 and 70 years, consistent with thymic production already being irrelevant in the 20s (Naylor et al., 2005). Similar to men, increased turnover rates in non-human primates have only been noted in relatively old animals, probably in response to critical lymphopenic thresholds (Cicin-Sain et al., 2007).

Homeostatic proliferation is therefore the major driving force of T cell generation in humans and generally sufficient to maintain sizable numbers of naïve T cells, in particular of CD4 T cells. It is, however, non-random and is subject to peripheral selection pressures that impose cumulative effects with progressive age (Table 1). Peripheral recognition of self MHC/peptide complexes provides necessary signals for naïve T cell survival. In murine lymphopenia models, accelerated homeostatic proliferation is associated with the selection of T cells recognizing self with higher affinity (Kassiotis et al., 2003; Kieper et al., 2004). Nikolich-Zugich and colleagues have studied the repertoire of CD8 T cells specific for foreign antigen in unprimed mice at different ages and have found a repertoire contraction with selection for antigen-specific T cells that presumably have been selected on self because they respond to lymphopenia with a higher proliferative rate than random T cells (Rudd et al., 2011). Evidence for repertoire skewing due to homeostatic maintenance has also been provided for the human system. CD4 T cells expressing the CD31 marker more frequently carry T cell receptor excision circle episomes than CD31-negative naïve CD4 T cells (Kimmig et al., 2002), suggesting a lesser replicative history. By comparing the repertoire of CD31-positive and CD31-negative naïve CD4 T cells, Thiel and colleagues clearly demonstrated a skewing of the repertoire in the CD31-negative population (Kohler et al., 2005). One possible outcome of cumulative homeostatic proliferation is the selection of a T cell receptor repertoire that is prone to autoreactivity (Goronzy and Weyand, 2001). In support of this finding, the naïve CD4 TCR repertoire of RA patients has been reported to differ in the frequencies of TCR Vβ–Jβ combinations compared to HLA-DRB1 matched healthy individuals (Walser-Kuntz et al., 1995). The age-associated repertoire skewing in these RA patients may be accelerated by increased T cell loss due to defective DNA repair mechanisms and compensatory increased peripheral replication leading to telomere shortening and TCR repertoire contraction (Goronzy et al., 2006).

**Table 1:** **Reshaping of the peripheral T cell repertoire in the aging host – a risk factor for autoimmunity**.

Homeostatic proliferation with selection for self recognition
Imbalance of pro- and anti-apoptotic molecules prolonging T cell survival
Cytokine-driven T cell expansion (IL-7, IL-15, IL-21) with selection for cytokine responsiveness
Lymphopenia

## Reduced T Cell Receptor Signaling Strength – A Risk Factor for Autoimmunity?

**Figure 2:**
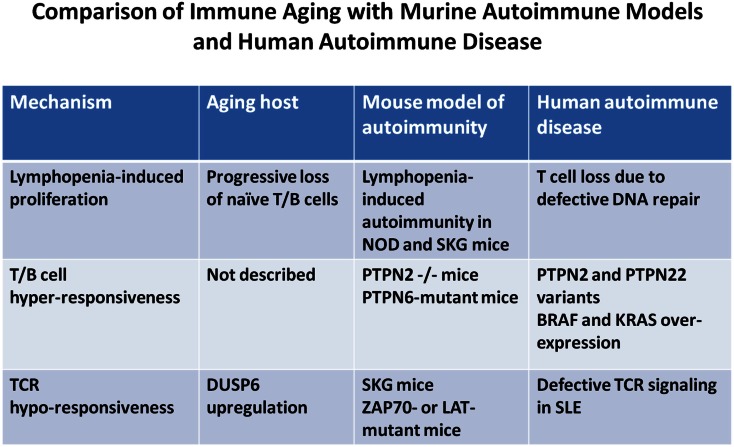
**Comparison of immune aging manifestations and autoimmune pathomechanisms**. The figure highlights mechanistic parallels between immune aging, animal models of autoimmune diseases, and human autoimmune diseases. Few selected animal models and human diseases representative of many others were chosen to illustrate the mechanistic path ways involved.

Intuitively, one would predict that hyperactivity of the TCR signaling pathway confers autoimmunity. Stimulation of the TCR initiates a cascade of tyrosine phosphorylation events that is regulated by an intricate network of tyrosine kinases and tyrosine phosphatases. Although both can have activating as well as inhibiting function depending on the phosphotyrosine targeted, quiescence is mainly regulated by phosphatase activity. Indeed, mutation or deletion of several phosphatases has been shown to cause autoimmunity (Zikherman and Weiss, 2009). A classic example is the moth-eaten mouse in which SHP-1 (PTPN6) is mutated (Shultz et al., 1997). Also, deletion of PTPN2 in T cells leads to spontaneous autoimmunity in mice (Wiede et al., 2011). Moreover, a PTPN2 variant is associated with type I diabetes mellitus, RA, and celiac disease in men (Consortium, 2007; Parkes et al., 2007; Franke et al., 2008). Similarly, a variant PTPN22 that undergoes faster degradation is associated with multiple human autoimmune diseases (Stanford et al., 2010; Zhang et al., 2011). Paradoxically, however, in several model systems autoimmunity is a result of decreased TCR signaling (Figure 2). The classical example is the SKG mouse in which a mutation of the ZAP70 gene dampens the activation-induced TCR signaling cascade; however, that mouse develops a Th17-mediated autoimmune disease that resembles features of RA (Hirota et al., 2007).

The pathogenesis of autoimmune disease in the SKG mouse remains unexplained. Positive, as well as negative, selection in the thymus appears to be influenced by the mutation (Sakaguchi et al., 2003; Hsu et al., 2009). The mutation impairs the association of ZAP70 with the T cell receptor zeta-chain leading to impaired TCR signaling induction in peripheral T cells and reduced proliferative responses after TCR stimulation. However, peripheral tolerance appears to be unstable. Germ-free SKG homozygous mice do not develop disease, but stimulation of pattern-recognition receptors induces onset of disease which is T cell-dependent (Yoshitomi et al., 2005). In contrast, SKG heterozygous mice develop spontaneous autoimmune disease emphasizing the impact of graded TCR stimulation (Tanaka et al., 2010). Autoimmune manifestations are also seen in ZAP70 hypomorphic mutants that allow the study of the impact of graded T cell receptor signaling strengths (Siggs et al., 2007). Mice with a partial, but not mice with a severe, defect in ZAP70 signaling developed increased Th2 polarization with the production of antinuclear antibodies. Similarly, an LAT mutation that inhibits PLCγ activation but leaves ERK phosphorylation intact, results in a multi-organ inflammatory disease with the production of antinuclear antibodies (Sommers et al., 2005; Genton et al., 2006). Overall, autoimmune disease mouse models with hyperactive TCR activation pathways appear to be the exception rather than the norm while several examples exist where selected changes of the TCR signaling complex that cause a reduced quantitative response or a shift in kinetics increases the risk for autoimmunity. While effects on thymic selection cannot be excluded as the driving pathomechanisms, there clearly needs to be a peripheral trigger. Peripheral tolerance mechanisms appear to be less stable in these mice. Presumably, the defect is associated with the establishment of a signaling equilibrium at rest that is less stable and more prone to non-linear transitions. Of note, signaling networks are in a constitutively active state in resting T cells and, therefore, stochastic fluctuation in positive signals or a drop in negative control pathways may be sufficient in such T cells that have adapted their networks to low signaling strengths.

The graded signaling defects in the ZAP70-mutated mice are very similar to the signaling defects seen with progressive aging. Elderly naïve CD4 T cells have increasing cytoplasmic concentrations of DUSP6. This increase is due to a decline in miRNA-181a that posttranscriptionally controls the expression of DUSP6 (Li et al., 2012). DUSP6 is a cytoplasmic phosphatase that selectively regulates the phosphorylation of ERK (Muda et al., 1996; Bettini and Kersh, 2007). Phosphorylated ERK is a critical regulator of setting the TCR’s threshold at which antigen recognition is translated into T cell activation. By serine phosphorylating Lck, active ERK prevents the recruitment of SHP-1 to the T cell signaling complex and therefore allows sustained signaling (Stefanova et al., 2003; Altan-Bonnet and Germain, 2005). Consequently, increased cytoplasmic concentrations of DUSP6 reduce the availability of phosphorylated ERK and increase the threshold of T lymphocytes to respond, in particular to low affinity antigens (Li et al., 2007). Overexpressing miRNA-181a or silencing DUSP6 restores T cell activation in old CD4 T cells (Li et al., 2012). Similar to DUSP6, PTPN7 controls proximal ERK phosphorylation after T cell activation and therefore the T cell receptor activation threshold (Saxena et al., 1999), however, it is currently unknown whether PTPN7 is subject to concentration changes with aging or cell differentiation as has been shown for DUSP6. How could a lesser ERK response and a heightened T cell receptor activation threshold lead to autoimmunity? It is possible that signaling networks adapt to a reduced input and that a unstable state of peripheral unresponsiveness is established which is more susceptible to spontaneous activation and interferes with tolerance maintenance similar to the ZAP70-mutated mice.

Evidence for aberrations in signaling networks has also been found in several human autoimmune diseases. Some, like the increased risk with a PTPN22 or PTPN2 variant are inherited as discussed above. Others may represent adaptations to acquired changes in signaling networks. Examples are the substitution of FcRγ for the CD3 zeta chain in SLE or the overexpression of ERK pathway members in RA (Tenbrock et al., 2007; Moulton and Tsokos, 2011).

In the previous section, we have argued that aging goes hand-in-hand with homeostatic maintenance mechanisms that eventually reshape the peripheral repertoire. This repertoire restructuring process not only impacts the selection of TCR, but is associated with profound functional changes as well (Table 1). Haynes and Swain have shown in mouse models that survival times for CD4 T cells increase with age due to the decreased expression of BIM and that this prolonged survival results in the acquisition of T cell defects (Tsukamoto et al., 2009). Since T cell homeostasis, in addition to the balance between pro-apoptotic and pro-survival molecules, is dependent upon TCR signaling and the homeostatic cytokines IL-7, IL-15, and IL-21, homeostatic maintenance and proliferation will eventually result in the selection of T cell clones that are optimized for their survival and growth behavior, regulated by both TCR and STAT signaling. Such selected clones should have compensated for some of the age-associated defects such as miRNA-181a loss and overexpression of DUSP6 and have recalibrated their signaling networks. In our *in silico* modeling of T cell homeostasis over lifetime, we have shown that cumulative inheritable changes in growth behavior can account for the abrupt contraction and the increased turnover that is seen in old age (Naylor et al., 2005; Johnson et al., 2012). A similar selection might also explain the age-associated increased incidence of autoimmune disease.

## Lymphopenia-Induced Autoimmunity and Homeostatic Cytokines

While homeostatic proliferation and maintenance, either in form of selection for a more autoreactive TCR repertoire or for general fitness to proliferate and survive, provides a model to explain the age-associated increased frequency of autoimmunity, lymphocyte proliferation itself appears to be associated with an increased risk, probably due to tolerance-abating signals from homeostatic cytokines. In several animal models, lymphopenia significantly contributes to the occurrence of autoimmunity (Hickman and Turka, 2005); the risk has generally been related to the activity of homeostatic cytokines (King et al., 2004; Calzascia et al., 2008). The NOD mouse, which is prone to develop autoimmune diabetes mellitus, is lymphopenic and the development of disease is dependent on IL-21-mediated turnover of islet-specific T cells (King et al., 2004). Similarly, in the model described by Calzascia et al. (2008) the presence of β-islet cell-specific self-reactive CD4 T cells was not sufficient to develop diabetes, however, such autoreactive T cells conferred disease when driven into proliferation by IL-7. Also, the SKG mouse model of RA described above is lymphopenic and characterized by increased homeostatic proliferation, possibly to compensate for defective thymic generation (own unpublished observation). This increased turnover in SKG mice is directly or indirectly linked to increased activation of the ERK pathways. Data from our lab have shown that even the slightest suppression of ERK activity in the steady-state delays disease onset suggesting that homeostatic proliferation is a facilitator of autoimmune manifestations (Singh et al., 2009).

**Figure 3:**
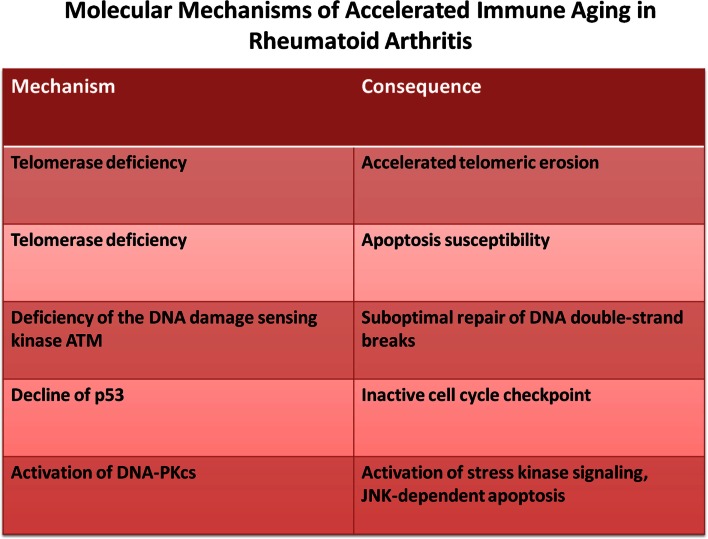
**Molecular mechanism of accelerated immune aging**. Rheumatoid arthritis is an autoimmune disease that is characterized by accelerated immune aging. The principle defects reside in impaired DNA repair responses.

While lymphocyte replenishment is a constant challenge throughout adult life, it appears to be steady and only increases late in the eighth decade (Wallace et al., 2004; Naylor et al., 2005). Increased homeostatic proliferation, therefore, does not appear to be relevant for developing autoimmune disease during normal healthy aging. However, several autoimmune diseases exhibit evidence of accelerated immune aging, mostly concluded from age-inappropriate telomeric erosion (Goronzy and Weyand, 2003; Goronzy et al., 2006; Georgin-Lavialle et al., 2010; Hohensinner et al., 2011). It is, therefore, possible that in these patients subclinical lymphopenia already develops at an earlier age which, in turn, enhances homeostatic turnover to set the stage for overt autoimmune manifestations (Goronzy et al., 2010). The best studied example is RA where increased replicative history is not only supported by the finding of telomeric erosion (Schonland et al., 2003; Fujii et al., 2009), but also by increased dilution of TCR excision circles (Koetz et al., 2000; Ponchel et al., 2002) and by repertoire contraction in TCR β-chain diversity within the naïve compartment (Wagner et al., 1998) and accumulation of terminally differentiated CD4 effector T cells that have lost the expression of CD28 (Schmidt et al., 1996a,b; Warrington et al., 2001). The underlying defect appears to be a dysfunctional DNA damage repair response that results in increased apoptosis and accelerated T cell loss. At least two defective pathways have been described so far. Reduced expression of telomerase in naïve RA T cells does not only lead to insufficient telomeric repair, but also to increased apoptotic susceptibility independent of telomeric lengths (Fujii et al., 2009). Second, reduced expression of ATM and other members of the ATM-MRE repair complex lead to insufficient DNA repair, chronic DNA damage responses and apoptosis (Shao et al., 2009). The increased loss may lead to the accumulation of end-differentiated cells that are relatively resistant to apoptosis (Schirmer et al., 1998; Vallejo et al., 2000). Altogether, these observations are consistent with the model that in RA increased cell loss and associated compensatory increased activity of homeostatic cytokines may be a facilitator of autoimmunity (Figure 3).

To directly address the question whether and how homeostatic cytokine signaling may affect tolerance mechanisms, we have examined whether exposure to homeostatic cytokines primes sensitivity to subsequent TCR stimulation (Deshpande et al., 2013). In these experiments, naïve CD4 T cells from healthy individuals were primed with increasing concentrations of IL-7, IL-15, or IL-21, followed by TCR stimulation in the absence of exogenous cytokines. Cytokine priming in general amplified TCR signals and enabled T cell activation in response to suboptimal stimulation. Of particular interest, cytokine priming of naïve CD4 T cells from HLA-DRB1*0401 healthy donors enabled proliferative responses to citrullinated vimentin and melanocyte glycoprotein gp100 peptides that have been previously shown to be relevant for the T cell responses in patients with RA (Hill et al., 2003; Law et al., 2012) and melanoma (Phan et al., 2003), respectively. The underlying mechanism is a PI3-kinase-dependent activation of RAS by the homeostatic cytokines that initiates a SOS-mediated amplification loop in ERK phosphorylation after TCR stimulation and is sufficient to overcome low-responsiveness to low affinity self-antigens (Deshpande et al., 2013). In conclusion, exposure to homeostatic cytokines transiently reduces the threshold of T cells to respond to low affinity self-antigens and could thereby initiate a program of proliferation and differentiation that leads to memory differentiation of autoreactive T cells.

## Stimulatory and Inhibitory NK Cell-Associated Receptors on Aging T Cells

One of the most striking findings in immune aging is a increased expression of regulatory cell surface receptors, mostly on end-differentiated CD8 T cells and to a lesser degree also on other CD4 and CD8 T cell subsets (Table 2). These receptors include killer immunoglobulin-like receptors (KIRs), killer cell lectin-like receptors (KLRs), and the immunoglobulin-like transcript (ILT/CD85) receptors, all typically associated with NK cell functions (Abedin et al., 2005; Cavanagh et al., 2011). Their expression is generally correlated with the loss of the classical costimulatory molecules CD27 and CD28 (Namekawa et al., 2000; Snyder et al., 2002; Ince et al., 2004; van Bergen et al., 2004). Although varying in fine specificity, KIRs and CD85/ILTs all recognize MHC class I molecules (Boyington et al., 2001; Trowsdale, 2001; Vilches and Parham, 2002). The C-type lectin receptors are more diverse in their ligand specificities. CD94/NKGs recognize MHC class I molecules (Lopez-Botet and Bellon, 1999), NKG2D ligands include a number of cellular stress-inducible molecules (Gonzalez et al., 2008) while KLRG1 binds to cadherin (Ito et al., 2006). These receptor-ligand pairs are fundamentally different from the traditional costimulatory or inhibitory receptors that fine-tune antigen-induced T cell activation. The ligands are constitutively expressed on cell types that are not specialized in antigen presentation. The expression of these regulatory receptors on T cells with aging therefore confers regulatory control to tissue-residing cells that usually do not participate in immune responses.

**Table 2:** **Mechanisms altering T cell receptor responsiveness with age**.

Loss of CD28
Acquisition of co-stimulatory receptors (KIR2DS/3DS, CD94/NKG2C, NKG2D)
*De novo* expression of co-inhibitory receptors (KIR, KLR, ILT2, PD1)
Rewiring of signaling cascades due to chronic cytokine stimulation
Rewiring of signaling cascades due to kinase or adaptor expressions (seen in RA and SLE, not yet shown in aging)
Rewiring of signaling cascades due to increased phosphatase expression (e.g., DUSP6)

The majority of these molecules have one or several ITIM signaling domains and function to recruit cytoplasmic phosphatases. Similar to their role in NK cells, they can dampen activation signals and are therefore thought to account for defective T cell responses in the elderly, a model reminiscent of T cell exhaustion conferred by PD1 (Wherry, 2011). However, TCR-induced activation of effector function such as cytotoxicity or cytokine secretion is hardly suppressed in aged T cells and is much less affected than proliferation (Henel et al., 2006; Henson et al., 2009). We have proposed that recruitment of phosphatase activity to the signaling complex may be late or incomplete; therefore, only inhibiting selected functions, in particular proliferation (Henel et al., 2006). In this model, expression of inhibitory receptors could be beneficial for the aging immune system. While T cells are still competent to respond to antigen recognition, clonal expansion is restricted protecting the host from undue skewing of the repertoire.

Increased frequencies of CD28-negative CD4 and CD8 T cells expressing NK cell-associated receptors are a common finding in autoimmune diseases, in particular rheumatoid arthritis (Yen et al., 2001; Nakajima et al., 2003; Snyder et al., 2003; Qin et al., 2011; Boita et al., 2012). While most of the receptors are inhibitory, some of them have stimulatory function, notably NKG2D, KIR2DL4, and the short isoforms of KIRs as well as CD94/NKG2C. Like in NK cells, the expression of different isoforms appears to be stochastic. This has been best shown for KIRs where expression on T cells and NK cells is entirely and exclusively dependent on promoter demethylation (Santourlidis et al., 2002; Chan et al., 2003; Li et al., 2008, 2009). We have analyzed the KIR expression pattern on the progeny of an *in vivo* expanded T cell clone identified by the identical T cell receptor (Snyder et al., 2002). The data were most consistent with the model that acquisition of different KIR isoform on each cell is successive and cumulative generating increasingly complex patterns. Expression of stimulatory receptors on T cells could therefore overcome tolerance or anergy. NKG2D has been implicated in several autoimmune diseases including celiac disease, type 1 diabetes mellitus, Crohn’s disease, and rheumatoid arthritis (Gonzalez et al., 2006; Van Belle and von Herrath, 2009). Dependent on the co-expression of its adaptor molecule DAP10, NKG2D can activate the PI3K-AKT pathway and thereby bypass costimulatory deficits (Billadeau et al., 2003). Stimulatory KIR molecules (e.g., KIR2DS1, KIR2DS2, etc.) require DAP12 to be fully functional (Wu et al., 2000). We have isolated KIR2DS2-DAP12-positive CD4 T cells from RA patients (Snyder et al., 2004a). Stimulation of KIR2DS2 in these T cells led to full activation without the need for TCR stimulation. Co-expression of a stimulatory KIR and DAP12 is infrequent, however, even KIR2DS2 in the absence of DAP12 can be expressed on the cell surface and then provides a costimulatory signal to TCR triggering (Snyder et al., 2004a,b). Stimulatory KIRs have been described as disease risk genes for RA and psoriasis, supporting a role for these receptors in autoimmunity (Yen et al., 2001; Martin et al., 2002). The KIR receptor KIR2DL4, while containing an ITIM motif in its cytoplasmic tail, can also provide stimulatory signals, but mostly at the level of the endosome (Rajagopalan and Long, 2012). When KIR2DL4 binds its ligand, HLA-G, the complex is internalized and activates DNA-PKcs and downstream PI3K (Rajagopalan, 2010; Rajagopalan et al., 2010).

In summary, NK cell-associated stimulatory receptors when expressed on aged T cells provide positive signals that are partially or even fully stimulatory and then activate the T cell even in the absence of antigen. Notably, relevant ligands are not restricted to APCs. As such, they can provide signals to T cells without the temporal and spatial control afforded by traditional costimulators.

## Signaling Pathways Induced by Chronic DNA Damage Responses

Telomeric erosion is an accepted index of aging and is found in several cell lineages including naïve and memory T cells (Hodes et al., 2002; Colmegna et al., 2008). Generally considered as a corollary of increased proliferation, changes in telomerase expression and failing DNA repair mechanisms appear to be at least equally contributory (Reed et al., 2004; Fujii et al., 2009; Effros, 2011; Hohensinner et al., 2011). Replicative attrition of telomeric ends activates DNA damage response pathways which can arrest cell division (Ciccia and Elledge, 2010; Hiom, 2010) but also skew the homeostasis of the cytoplasmic and nuclear signaling networks in the absence of extrinsic stimuli (Rube et al., 2011; Armanios, 2013). Telomeric erosion has been found in many autoimmune diseases where it may not only be a consequence of the inflammatory disease, but also contribute to disease development and manifestation. This concept has been best studied in patients with rheumatoid arthritis who have evidence of accelerated immune aging by about 20 years (Goronzy et al., 2010). In these patients, cells express reduced telomerase activity which causes telomeric erosion in all hematopoietic lineages ranging from stem cells to mature naïve and memory T cells (Fujii et al., 2009). Increased DNA damage is not limited to the telomeres, RA patients have also evidence of DNA double-strand breaks compare to age-matched healthy controls as determined by comet assay (Shao et al., 2009). DNA damage is more prevalent in memory T cells where it gradually increases with age. In contrast, naïve T cells in healthy individuals have little DNA damage up to the age of 70 years when it starts to increase (Shao et al., 2009). At any age and in both T cell subsets, DNA damage is increased in RA patients. The underlying mechanism is a reduced expression and function of the DNA damage sensing kinase ATM and other members of the ATM-MRE pathway.

Chronic DNA damage responses play a major role in regulating intrinsic cell activation and in particular the production of inflammatory mediators following cellular senescence (Rodier et al., 2009). Rodier and Campisi (2011) have described the senescence-associated secretory phenotype (SASP) in senescent fibroblasts which may be applicable to other cell types as well. SASP is dependent on DNA damage response signaling involving the DNA repair molecules ATM, NBS1, and CHK2. p53 activation is a negative regulator of SASP, but can be overcome in the context of DNA damage responses by p38/NFκB (Freund et al., 2011; Gudkov et al., 2011). SASP-mediated production of proinflammatory cytokines is sensitive to the suppressive action of glucocorticoids, which, however, cannot revert the senescence-associated growth arrest (Laberge et al., 2012).

A second pathway through which chronic DNA damage responses reprogram cells toward proinflammatory effector functions is the activation of DNA-PKcs. T cells from patients with RA have increased DNA-PKcs activity, possibly as a consequence of ATM deficiency and associated increased DNA damage (Shao et al., 2009, 2010). DNA-PKcs influences intracellular signaling pathways through at least two mechanisms. It activates the inflammasome and increases NFκB activity; and it activates the stress kinase JNK pathway (Rajagopalan et al., 2010; Shao et al., 2010). Both pathways can contribute to the production of inflammatory cytokines.

## Conclusion

The pathomechanisms of autoimmunity are as multifaceted as the manifestations of immune aging. Although both processes appear to be contradictory at first sight, there are many parallels. In particular, impaired immune effectiveness, a hallmark of immune aging, mirrors many of the conditions in animal models that render mice susceptible to develop an autoimmune disease. Moreover, it is a well-known clinical experience that patients with many inheritable immune deficiency diseases are prone to also develop an autoimmune disease. It is not the responsiveness of the immune system, but its lack of stability that predisposes for tolerance failure. The aging immune system, through its attempt to endure and to compensate for age-associated defects is acquiring an unstable state. An increased risk for autoimmunity may be the price we have to pay to preserve some immune function into older age.

## Conflict of Interest Statement

The authors declare that the research was conducted in the absence of any commercial or financial relationships that could be construed as a potential conflict of interest.
